# Gastroduodenal Intussusception in Peutz-Jeghers Syndrome

**DOI:** 10.5334/jbsr.2572

**Published:** 2022-01-19

**Authors:** Ayoub Mokhtari, Alessandro De Leucio, Grammatina Boitsios

**Affiliations:** 1 Université Libre de Bruxelles (ULB), BE; 2 Hôpital Universitaire des Enfants Reine Fabiola, BE

**Keywords:** Peutz-Jeghers syndrome (PJS), gastroduodenal intussusception, CT

## Abstract

**Teaching points**: Gastroduodenal intussusception is an infrequent cause of abdominal pain in children, for which a lead-point is nearly ubiquitous, which imposes endoscopic reduction as the first line of treatment.

## Case History

**Figure 1:**
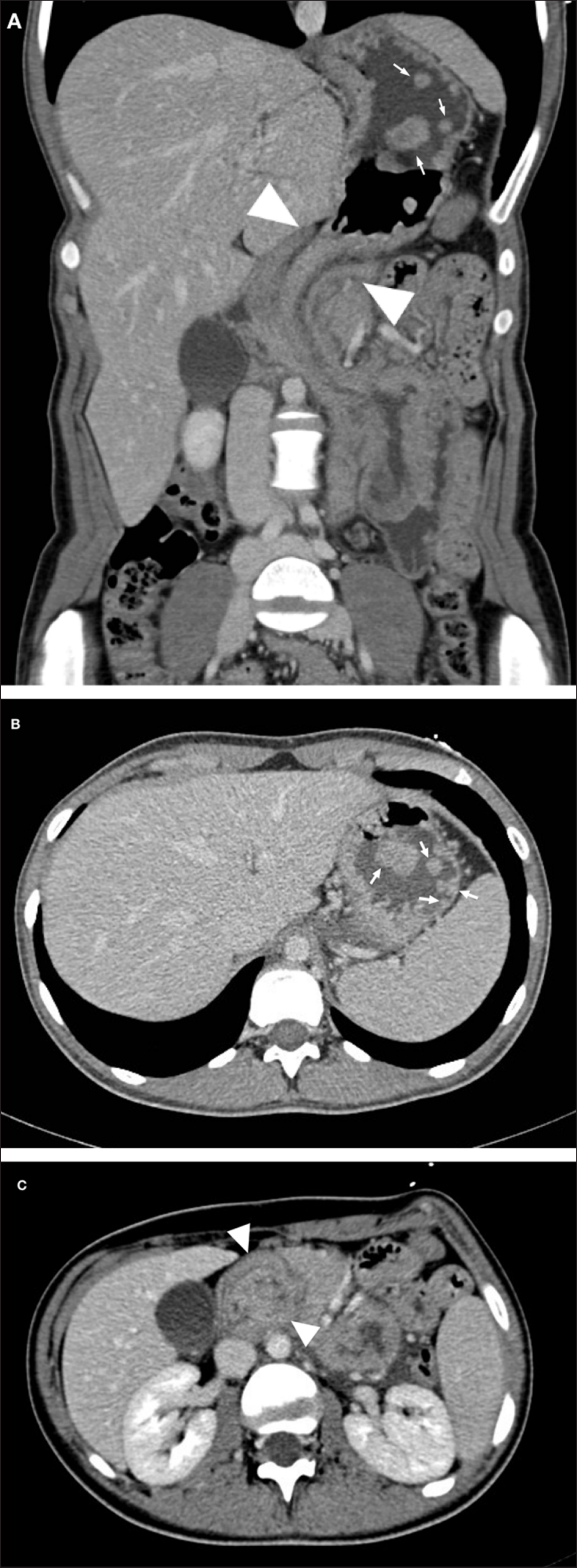


A 16-year-old girl presented in the emergency department afebrile with hypogastric abdominal pain, nausea, and vomiting. The patient was known to have Peutz-Jeghers syndrome (PJS) with multiple hamartomatous gastrointestinal polyps since the histological analysis of a 50 cm small bowel resection, which occurred at the age of four in the course of acute ileo-ileal intussusception. A contrast-enhanced abdominal computed tomography (CT) on coronal curved reformation through the stomach and duodenal axes (***Figure 1a***), in the axial plane at the level of the stomach body (***Figure 1b***), and the pancreatic head (***Figure 1c***) showed multiple enhancing gastric polyps (arrows), along with intussusception (arrowheads) of the stomach within the proximal portion of the duodenum. There was no compromise of the gastroduodenal wall enhancement. The CT findings were confirmed on subsequent gastroduodenal endoscopy carried out for air insufflation, resulting in successful reduction of the intussusception.

## Commentary

The most common polyposis leading to gastroduodenal intussusception is the PJS, an autosomal dominant disease caused by a mutation on the genes STK1. PJS is characterized by hamartomatous polyposis in the gastrointestinal tract (predominantly in the small bowel) resulting in gastroduodenal and ileo-ileal intussusception, and mucocutaneous pigmentation predominantly on the lips and around the mouth. The diagnosis of PJS is based on histological analysis of the polyps, family history of PJS, or mucocutaneous lesions [1].

Patients with PJS present with recurrent and variable degrees of abdominal pain due to intestinal occlusion, infarction, or gastrointestinal bleeding caused by the endoluminal polyps [1]. Our patient presented with multiple episodes of intussusception during her childhood and in some of these episodes, small bowel resection was needed.

Ultrasound is the first modality of choice when assessing abdominal pain in PJS [1]. In our case, contrast-enhanced CT was preferred because of the clinical suspicion of bowel obstruction caused by band adhesions from previous surgeries. In addition, gastric assessment by ultrasound in case of gastroduodenal intussusception can be sometimes challenging due to the intestinal meteorism, and CT better depicts the extent of the intussusception and its repercussions such as bowel necrosis and obstruction, especially in older infants/teenagers.

As 95% of intussusception occurs in the small bowel, gastroduodenal intussusception is an extremely rare occurrence, even in patients with PJS [1]. An underlying lead point must be excluded, preferably via gastroduodenal endoscopy, which also allows the reduction of the intussusception by insufflation, avoiding surgery as in our case.
